# Dynamic contrast-enhanced magnetic resonance imaging of the wrist in children with juvenile idiopathic arthritis

**DOI:** 10.1007/s00247-016-3736-2

**Published:** 2016-12-12

**Authors:** Charlotte M. Nusman, Cristina Lavini, Robert Hemke, Matthan W. A. Caan, Dieneke Schonenberg-Meinema, Koert M. Dolman, Marion A. J. van Rossum, J. Merlijn van den Berg, Taco W. Kuijpers, Mario Maas

**Affiliations:** 1 Department of Pediatric Hematology, Immunology, Rheumatology and Infectious Disease, Academic Medical Center, Emma Children’s Hospital, Meibergdreef 9, 1105 AZ Amsterdam, The Netherlands; 2 Department of Radiology, Academic Medical Center, Meibergdreef 9, 1105 AZ Amsterdam, The Netherlands; 3 Department of Pediatrics, Sint Lucas Andreas Hospital, Jan Tooropstraat 164, 1061 AE Amsterdam, The Netherlands; 4 Department of Pediatric Rheumatology, Reade Institute location Jan van Breemen, Doctor Jan van Breemenstraat 2, 1056 AB Amsterdam, The Netherlands; 5 Department of Pediatrics, Academic Medical Center, Emma Children’s Hospital, Meibergdreef 9, 1105 AZ Amsterdam, The Netherlands

**Keywords:** Children, Dynamic contrast-enhanced magnetic resonance imaging, Juvenile idiopathic arthritis, Magnetic resonance imaging, Synovium, Wrist

## Abstract

**Background:**

Dynamic contrast-enhanced MRI provides information on the heterogeneity of the synovium, the primary target of disease in children with juvenile idiopathic arthritis (JIA).

**Objective:**

To evaluate the feasibility of dynamic contrast-enhanced MRI in the wrist of children with JIA using conventional descriptive measures and time-intensity-curve shape analysis. To explore the association between enhancement characteristics and clinical disease status.

**Materials and methods:**

Thirty-two children with JIA and wrist involvement underwent dynamic contrast-enhanced MRI with movement-registration and were classified using validated criteria as clinically active (*n* = 27) or inactive (*n* = 5). Outcome measures included descriptive parameters and the classification into time-intensity-curve shapes, which represent the patterns of signal intensity change over time. Differences in dynamic contrast-enhanced MRI outcome measures between clinically active and clinically inactive disease were analyzed and correlation with the Juvenile Arthritis Disease Activity Score was determined.

**Results:**

Comprehensive evaluation of disease status was technically feasible and the quality of the dynamic dataset was improved by movement registration. The conventional descriptive measure maximum enhancement differed significantly between clinically active and inactive disease (*P* = 0.019), whereas time-intensity-curve shape analysis showed no differences. Juvenile Arthritis Disease Activity Score correlated moderately with enhancing volume (*P* = 0.484).

**Conclusion:**

Dynamic contrast-enhanced MRI is a promising biomarker for evaluating disease status in children with JIA and wrist involvement. Conventional descriptive dynamic contrast-enhanced MRI measures are better associated with clinically active disease than time-intensity-curve shape analysis.

**Electronic supplementary material:**

The online version of this article (doi:10.1007/s00247-016-3736-2) contains supplementary material, which is available to authorized users.

## Introduction

Juvenile idiopathic arthritis (JIA) is a chronic joint disorder in children with the synovium as the main target tissue for inflammation. The synovitis can eventually lead to structural damage in children with JIA, which obviously goes hand in hand with increased morbidity and decreased quality of life. JIA has a relapsing-and-remitting disease course, making adequate assessment of disease activity a real challenge and necessity, in order to provide tailored treatment for every patient. Currently, the distinction between active and inactive disease is based on a clinical assessment, but imaging techniques are becoming more and more valuable in this respect.

Conventional contrast-enhanced MRI is the preferred imaging modality for detecing and monitoring disease activity in children with JIA [1]. In order to depict the synovium as primary disease target with more inflammation-specific MRI techniques, dynamic contrast-enhanced MRI was proposed in the field of arthritic diseases [2]. Dynamic contrast-enhanced MRI enables the time-dependent registration of changes in the MRI signal of the synovium after administration of intravenous contrast agent. The dynamic enhancement patterns of the synovial tissue yield information on tissue permeability and microvascularization. The differences in these patterns provide information on the biological heterogeneity of the synovium. This can be valuable for both diagnostic and prognostic purposes in the management of arthritic disease [3, 4].

Despite many efforts to increase the use of MRI to assess the inflamed wrist, imaging the wrist remains a serious challenge in the field of JIA. With the wrist being the most affected location in rheumatoid arthritis, previous studies on (dynamic contrast-enhanced) MRI in the adult wrist provide a good starting point for further evaluation in JIA [3, 5–20] (Supplementary file 1). In the literature, both quantitative and semiquantitative outcome measures are used for dynamic contrast-enhanced MRI, ranging from pharmacokinetic measures to conventional descriptive parameters and time-intensity-curve shape analysis. Conventional descriptive measures give information on the range and magnitude of the enhancement. Time-intensity-curve shape analysis includes a classification of the patterns of change in signal intensity over time into different classes [10, 21, 22]. Based on the experience with dynamic contrast-enhanced MRI in the knee of JIA and rheumatoid arthritis patients, a difference was expected between clinically active and inactive patients, with a dynamic enhancement pattern characterized by a fast initial enhancement and fast outflow of contrast agent in clinically active disease. This enhancement pattern points to enhanced vascularization and can be therefore be described as an aggressive pattern [23–25].

The objective of this study was to perform a comprehensive feasibility evaluation of dynamic contrast-enhanced MRI in the wrists of children with JIA using two types of outcome measures: conventional descriptive measures and time-intensity-curve shape analysis including movement registration. Secondly, we aimed to explore the association between enhancement characteristics and clinical disease status.

## Materials and methods

### Patients

Between March 2012 and July 2013, 32 consecutive children with JIA were prospectively included. Patients were recruited from one of three tertiary outpatient clinics. Institutional Review Board approval was obtained and informed consent was acquired from all parents, as well as from patients older than 12 years of age.

Three sets of criteria were used to classify the patients: diagnosis of the JIA subtype was made based on the International League of Associations for Rheumatology criteria (e.g., oligoarticular persistent, enthesitis-related arthritis), binary classification of disease activity was made based on the Wallace criteria (i.e. clinically active and clinically inactive) and a quantification on a continuous scale of disease activity was performed by means of the Juvenile Arthritis Disease Activity Score [26–28].

All patients either had current wrist involvement (clinically active) or previous wrist involvement (clinically inactive). The indication to perform an MRI was always clinically driven. For example, in the case of children with clinically active JIA, the MRI was made to confirm synovial inflammation in the wrist. An example of MRI indication in a clinically inactive patient was to evaluate whether treatment could be downscaled in case of absent synovial inflammation on MRI.

Exclusion criteria were a history of intra-articular corticosteroid injection in the wrist within the last 6 months, the need for anesthesia during the MRI examination and general contraindications for MRI.

### Clinical assessment

Clinical assessment was performed by one of the experienced pediatric rheumatologists (JMvdB 12 years, DS 5 years, KD 15 years, MvR >20 years) and consisted of a 67 active joint count defining presence of swelling, pain on motion/tenderness and limited range of motion. Visual analogue scales (100 mm) were used for the physician’s global assessment of overall disease activity, patient’s global assessment of overall well-being and a patient’s pain assessment. The Childhood Health Assessment Questionnaire was used to evaluate patients’ functional abilities [29]. Laboratory tests included erythrocyte sedimentation rate and C-reactive protein level. Clinical disease activity was reflected as a continuous variable by the Juvenile Arthritis Disease Activity Score, which ranges from 0 to 40 and is calculated by the sum of the following clinical variables: the physician and parent/patient global assessment of disease activity (both ranging from 0 to 10), reduced 10-active joint count and a normalized value of erythrocyte sedimentation rate to a 0-10 scale [28].

### MRI protocol

MRI was performed using an open-bore 1.0-Tesla MRI scanner (Panorama HFO; Philips Medical Systems, Best, The Netherlands) and a Sense wrist coil (Philips Medical Systems, Best, The Netherlands, 3 channels) with the child in a supine position with the arm along the side of the body [30]. No sedation was used. The most clinically affected wrist (present for the active patients or previous for the inactive patients) was imaged. The standard MRI protocol for the JIA wrist included the following sequences: before injection of intravenous contrast, a coronal short-tau inversion recovery sequence; coronal T1-weighted sequence, axial T1-weighted sequence and axial T2-weighted sequence with fat saturation. After the dynamic contrast-enhanced MRI (performed during injection of intravenous contrast agent), a coronal T1-weighted sequence and axial T1-weighted sequence with fat saturation were performed [31]. The dynamic contrast-enhanced MRI sequence was a 3-D T1-weighted fast field echo dynamic sequence, consisting of 28 dynamic scans and 31 slices per scan, with a temporal resolution of 15.5 s. Furthermore, the dynamic contrast-enhanced MRI sequence had an in-plane field of view of 80 × 100 mm and a slab thickness of 46.5 mm, with voxel size 1.0 × 1.0 mm × 1.5 mm, repetition time of 9.9 ms and an echo time of 6.9 ms. After 3 dynamic scans, intravenous contrast agent (0.1 mmol/kg bodyweight of Gadovist; Bayer Schering Pharma, Berlin, Germany) followed by a chase of 15 ml saline solution was injected through a 22-gauge needle with an injection speed of 3 ml/s by an automated injection device (Medrad, Warrendale, PA).

### Dynamic contrast-enhanced MRI analysis

In-house written codes developed in Matlab (Mathworks, Natick, MA) were used for movement registration, drawing of region of interests and extraction of the dynamic contrast-enhanced MRI parameters. We performed non-rigid registration of all dynamic volumes to correct for possible small, residual movements of the wrist. For this movement registration, we adopted a Discrete Cosine Transformation model [32] with two subsequent cut-off bases of 50 and 25 mm to allow for global and local convergence.

**Fig. 1:**
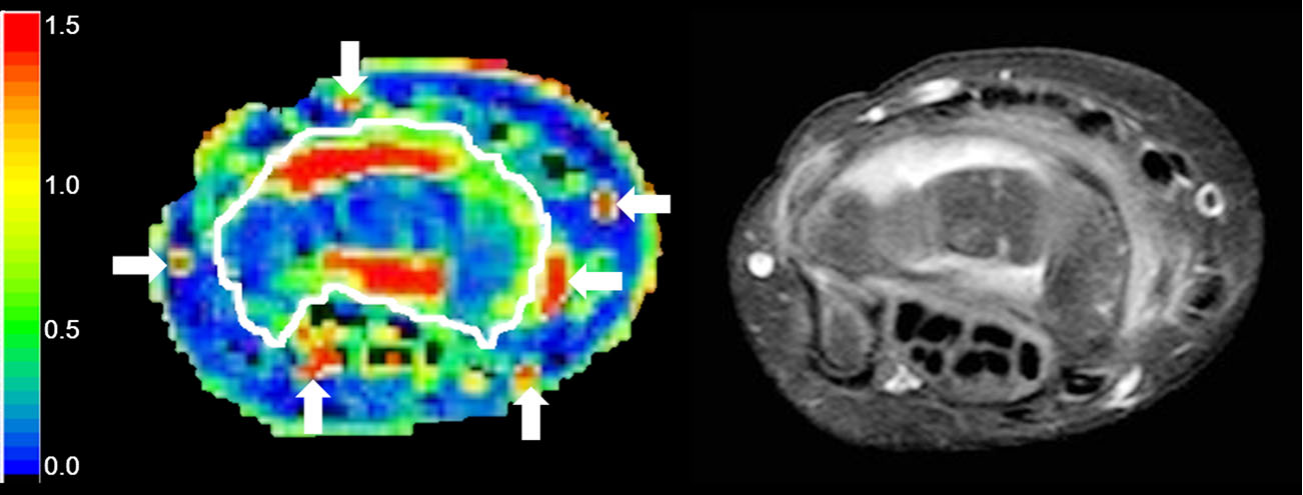
Example of a manually drawn region of interest (*white line*) on the maximal enhancement map (*left*) based on axial wrist MRI in a 9-year-old female with clinically active juvenile idiopathic arthritis. The *arrows* indicate the arteries and vessels, which have not been included in the region of interest. A high spatial resolution T1-weighted image with fat saturation after intravenous contrast administration (*right*) is added for anatomical reference

A large anatomy-based region of interest was manually drawn on the axial maximal enhancement maps by a radiology trainee (3 years of experience with imaging in JIA), while using the axial post-contrast T1-weighted dynamic contrast-enhanced MR image as a reference. The region of interest was drawn to exclude visible vessels, tendons and the epiphysis of the radius and ulna (Fig. 1) because no synovium was expected. The three most proximal and three most distal slices of the dynamic contrast-enhanced MRI suffered from fold-over artifacts and were therefore excluded from the analysis. To maintain a consistent field of view in every patient, the 20 slices distal to the last slice on which the ulna appeared were considered suitable for analysis of dynamic contrast-enhanced MRI.

**Fig. 2:**
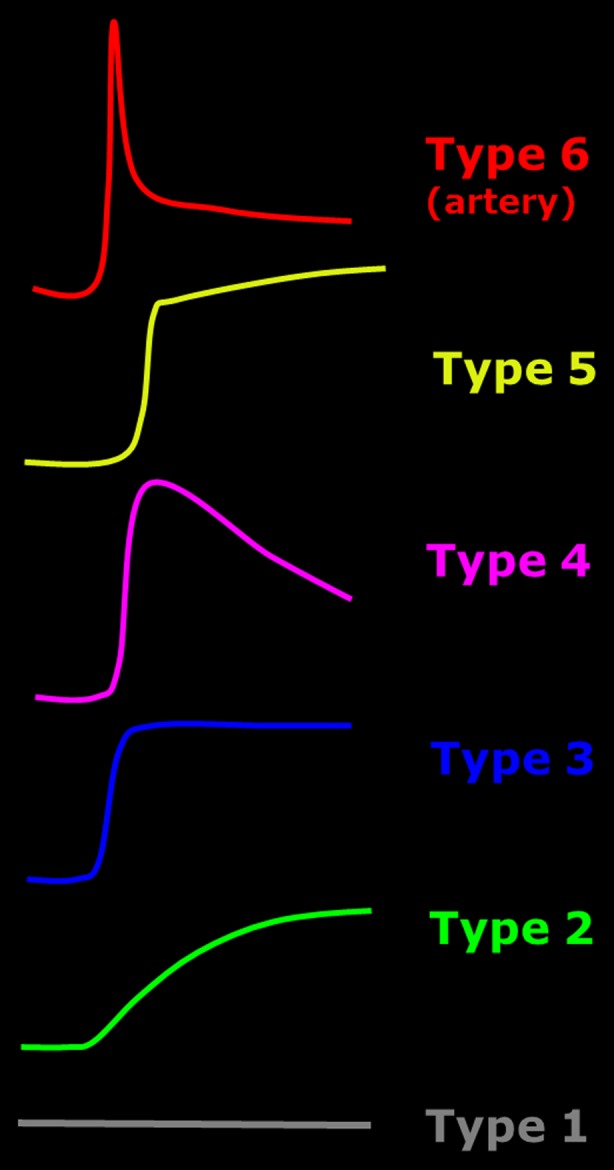
Explanation of the time-intensity-curve shapes and their color coding. Time-intensity-curve shape 7 is not shown as this class includes all the various shapes that cannot be classified as 1-6. Examples are given in [21]

The dynamic contrast-enhanced MR images were analyzed with in-house developed software, running on Matlab [33]. Outcome measures were divided in time-intensity-curve shapes and conventional descriptive measures. Time-intensity-curve shapes represent the change in signal intensity per voxel over time. Although time-intensity-curve shapes do not provide quantitative measures, indicate tissue characteristics such as degree of vascularization, tissue viability, perfusion and volume of the interstitial space. Every voxel within the region of interest is classified independently in one of the following seven time-intensity-curve shapes with unique colors: non-enhancing (1, gray), slow enhancing (2, green), fast enhancing followed by either a plateau phase (3, blue), washout phase (4, purple) or gradual increase (5, yellow), arterial patterns (6, red) and all remaining patterns (7, white) (Fig. 2). The classification algorithm is described in detail in [21]. The time-intensity-curve shapes were then analyzed based on their relative frequency in the drawn region of interest: the number of every time-intensity-curve shape was calculated by dividing the absolute number of pixels presenting a specific time-intensity-curve shape type by the absolute total number of time-intensity-curve shapes 2-7 (i.e. all the enhancing pixels). Only time-intensity-curve shapes 2-5 were used for statistical analysis, because time-intensity-curve shapes 1, 6 and 7 are not relevant for disease activity in JIA.

While bones of the wrist joint were included in the region of interest, they do not contribute to the final result as the bone does not enhance on dynamic contrast-enhanced MRI and all the results were calculated only on enhancing voxels (types 2 to 7).

Conventional descriptive measures included the median value of maximum enhancement (maximum enhancement of all enhancing voxels within the region of interest with time-intensity-curve shapes 2-7), median maximum initial slope (maximum slope of increase of all enhancing voxels within the region of interest with time-intensity-curve shapes 2-7), median initial area under the curve (area under the enhancement curve between start of injection and 90 s after injection) and the enhancing volume (total volume in millimeters of all enhancing voxels within the region of interest with time-intensity-curve shapes 2-7).

### Statistical analysis

Descriptive statistics were reported in terms of percentages means, medians, standard deviations and interquartile ranges. Normality plots were used to assess the normal distribution of the data. The Mann-Whitney *U* test was used to assess differences between clinical subgroups. A *P*-value <0.05 was considered statistically significant. A Spearman’s ρ correlation coefficient was used to assess the association between Juvenile Arthritis Disease Activity Score and dynamic contrast-enhanced MRI measures. Spearman’s ρ was interpreted as follows: <0.19 very weak, 0.20-0.39 weak, 0.40-0.59 moderate, 0.60-0.79 strong and >0.80 very strong. All statistical analyses were performed using SPSS version 20.0 (IBM SPSS, Chicago, IL).

## Results

### Patients

Thirty-two children with JIA were included (range: 8.2-17.9 years, 28.1% male). The JIA subtypes were as follows: 6 (19%) persistent oligoarthritis, 4 (13%) extended oligoarthritis, 15 (47%) polyarthritis rheumatoid factor negative, 2 (6%) polyarticular rheumatoid factor positive, 2 (6%) psoriatic arthritis, 2 (6%) enthesitis-related arthritis and 1 (3%) undifferentiated arthritis. The clinical characteristics of the patients divided per subgroup are summarized in Table 1.

**Table 1:** Clinical characteristics of 32 children with juvenile idiopathic arthritis

	Clinically active (*n* = 20)	Clinically inactive (*n* = 3)
Female	74.1	60
Age at MRI, mean (standard deviation), years	13.0 (2.6)	15.2 (1.1)
Disease duration at MRI, years	2.3 (1.4-6.5)	6.5 (2.7-9.6)
Number of actively inflamed joints	3 (1-5)	0 (0)
Number of joints with limitation of movement	2 (1-4)	1 (0-2)
Physician’s global visual analogue scale, mm	23 (12-38)	0 (0-0)
Patient’s overall well-being visual analogue scale, mm	40 (5-60)	0 (0-16)
Patient’s pain visual analogue scale, mm	44 (6-71)	0 (0-2)
Childhood health assessment questionnaire	1.00 (0.25-1.375)	0.375 (0-0.5)
Juvenile arthritis disease activity score (version 10)	8 (6-16)	0 (0-2)
Erythrocyte sedimentation rate, mm/h	6.5 (4.3-26.3)	3.5 (2.0-5.0)
C-reactive protein, mg/l	1.5 (0.3-7.0)	0.3 (0.3-0.9)

Unless indicated otherwise, values are given as the median (interquartile range)

### Dynamic contrast-enhanced MRI

All children with JIA underwent the dynamic contrast-enhanced MRI without difficulty: the added scan time was negligible in comparison to the complete duration of the MRI. Dynamic contrast-enhanced MRI yielded images with good quality and without appreciable movement artifacts. The motion correction was applied to address small shifts and rotations in the position of the hand between subsequent dynamic scans.

**Fig. 3:**
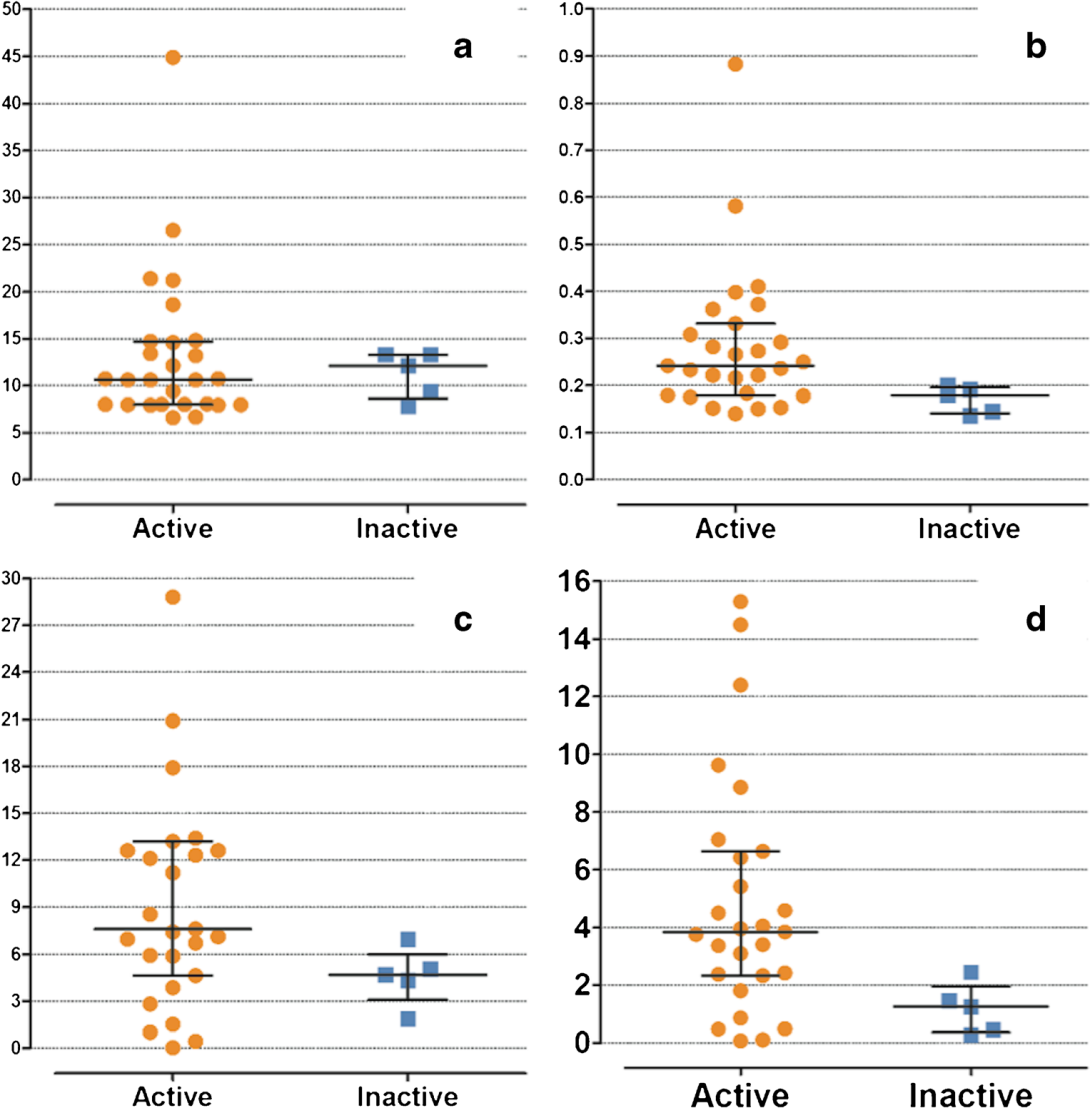
Boxplots show the difference in descriptive dynamic contrast-enhanced MRI measures between clinically active (*orange dots*) and inactive (*blue squares*) children with juvenile idiopathic arthritis. **a** Maximum initial slope (*P* = 0.880), (**b)** maximum enhancement, (*P* = 0.019), (**c)** initial area under the curve (*P* = 0.087) and (**d)** enhancing volume (*P* = 0.104). In (**c**), two data points of the active patients were outside the values of the y-axis (*y* = 60 and *y* = 123, respectively)

Among the conventional descriptive measures, maximum enhancement differed significantly between the clinically active (median maximum enhancement: 0.242) and clinically inactive (median maximum enhancement: 0.179) groups (*P* = 0.019). The other conventional descriptive dynamic contrast-enhanced MRI measures did not show any statistical significant differences between children with active and inactive disease (Fig. 3).

**Fig. 4:**
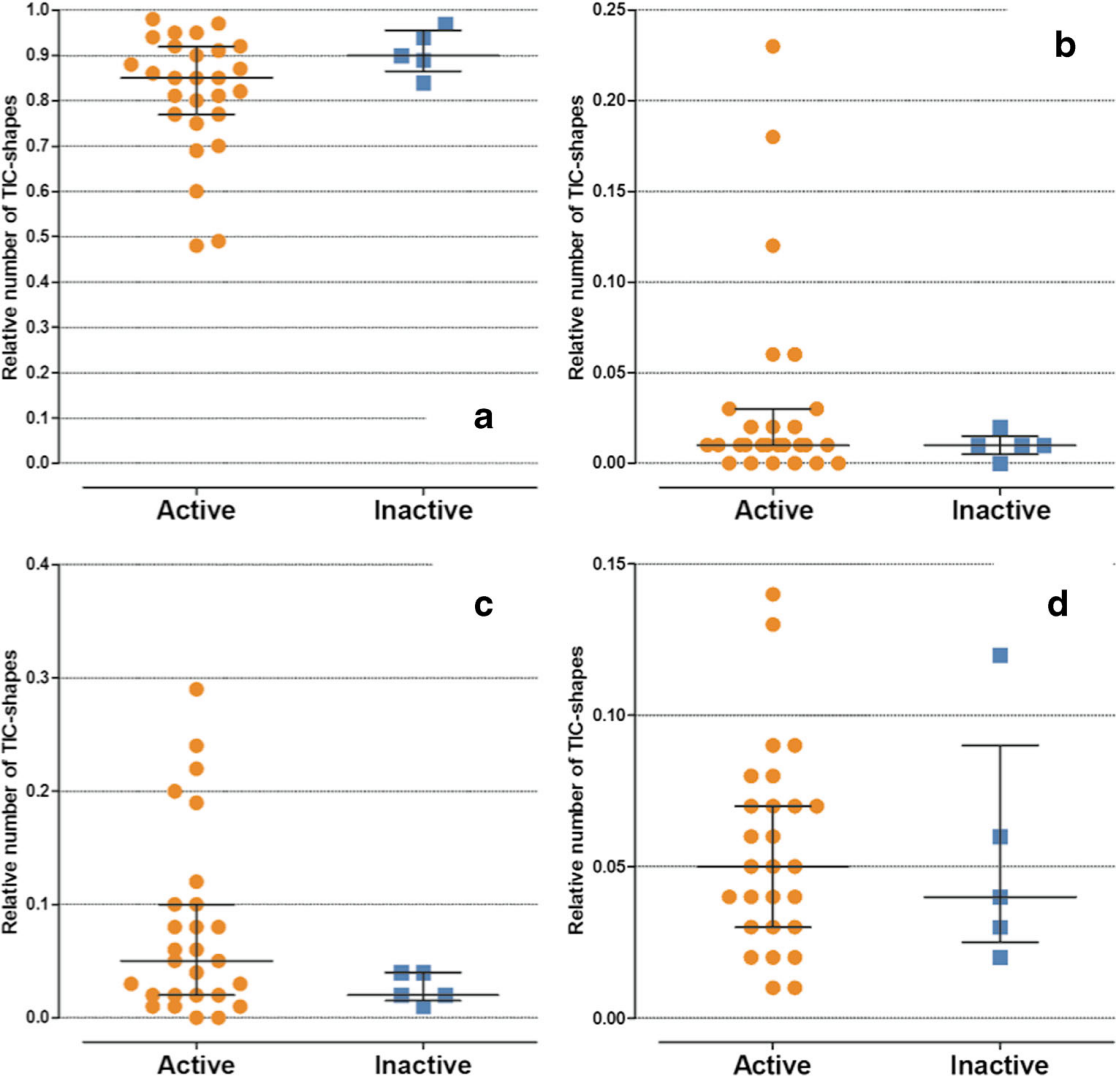
Boxplots show the difference in relative frequencies of time-intensity-curve shapes (y-axis) between children with clinically active (*orange dots*) and inactive (*blue squares*) juvenile idiopathic arthritis. **a** Time-intensity-curve shape type 2 (*P* = 0.150), (**b)** time-intensity-curve shape type 3 (*P* = 0.310), (**c)** time-intensity-curve shape type 4 (*P* = 0.166) and (**d)** time-intensity-curve shape type 5 (*P* = 0.725)

**Fig. 5:**
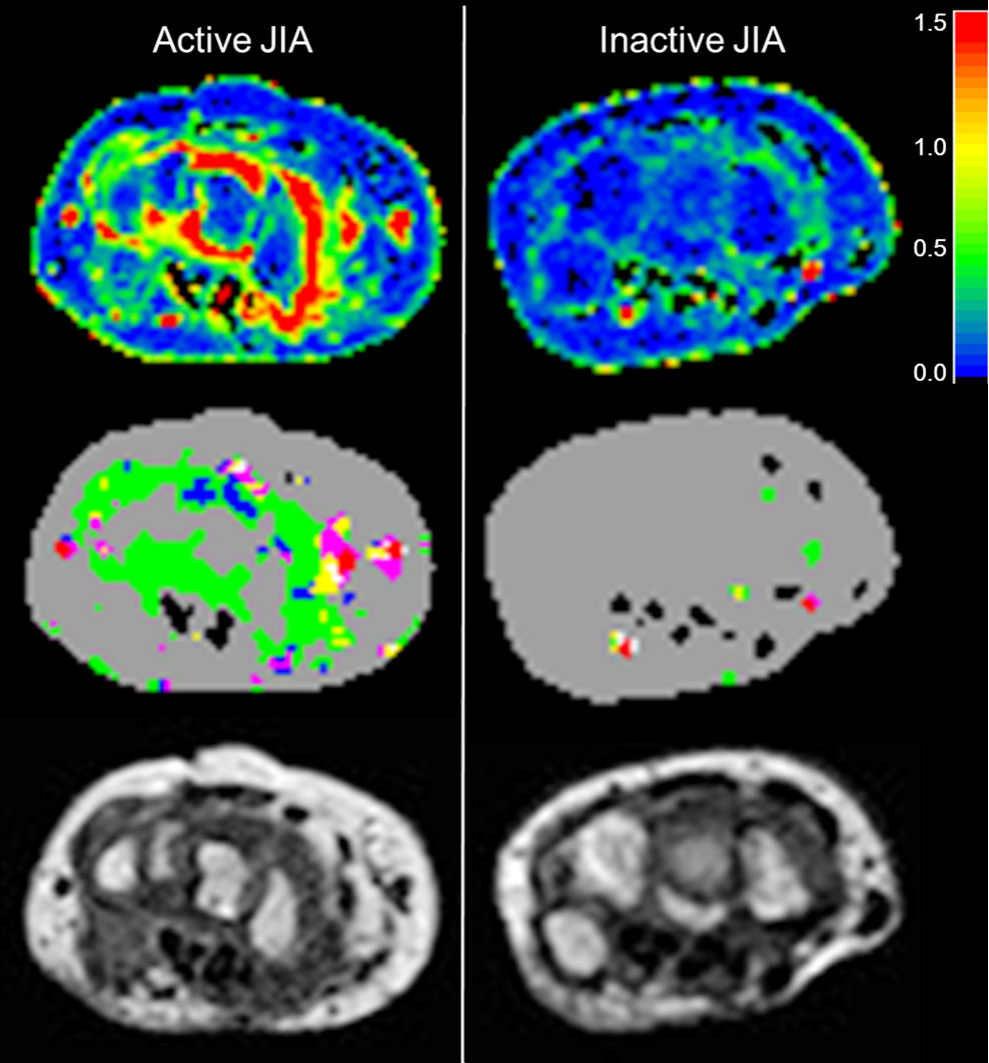
The difference between clinically active juvenile idiopathic arthritis in an 8-year-old boy (*left*) and inactive juvenile idiopathic arthritis in a 13-year-old girl (*right*) on maximum enhancement maps (*upper row*), time-intensity-curve shape maps (*middle row*), and T1-weighted dynamic contrast-enhanced-maps (*bottom row*). The colors of the time-intensity-curve shapes correspond with the time-intensity-curve shapes as mentioned in Fig. 2. Scan parameters: axial plane, echo time 6.9 ms, repetition time 9.9 ms, voxel size 1.0 mm × 1.0 mm × 1.5 mm, temporal resolution 15.5 s

The relative frequency of time-intensity-curve shape 2 tended to be higher in children with inactive disease; however, this difference was not statistically significant (*P* = 0.150). The children with active disease showed a trend toward higher numbers of time-intensity-curve shapes 3 and 4 compared to the children with inactive disease, but did not reach statistical significance (*P* = 0.310 and *P* = 0.166). No difference between active and inactive disease was found with respect to the number of time-intensity-curve shape 5 (Figs. 4 and 5).

Enhancing volume was the dynamic contrast-enhanced MRI measure that correlated best (moderate) with the clinical disease activity as reflected by Juvenile Arthritis Disease Activity Score (Table 2).

**Table 2:** Spearman correlation (ρ) of the dynamic contrast-enhanced MRI measures with Juvenile Arthritis Disease Activity Score-40 in 32 children with JIA

	ρ
Maximum enhancement	0.372
Enhancing volume	0.484
Initial area under the curve	0.326
Maximum initial slope	0.053
Time-intensity-curve shape 2	-0.089
Time-intensity-curve shape 3	0.379
Time-intensity-curve shape 4	0.249
Time-intensity-curve shape 5	-0.178

## Discussion

In the current study, we evaluated dynamic contrast-enhanced MRI in children with JIA and wrist involvement, by using both conventional descriptive measures and time-intensity-curve shape analysis. Dynamic contrast-enhanced MRI of the wrist is considered feasible, as the children with JIA could lie in the scanner for the time required for the investigation and image quality of both conventional descriptive measures and time-intensity-curve shape analysis was good. The descriptive dynamic contrast-enhanced MRI parameter maximum enhancement differed significantly between clinically active and inactive JIA disease. No significant larger number of the expected aggressive enhancement patterns (higher relative frequency of time-intensity-curve shapes 3 and 4) in children with clinically active disease could be proven, though a trend toward larger relative frequency of these types was observed.

In 2010, the first step towards the use of dynamic contrast-enhanced MRI of children with JIA was made through the assessment of its reliability and construct validity in a pilot study of 12 patients with wrist involvement [13]. The current study adds to the previous work by describing the differences in dynamic contrast-enhanced MRI measures in two clinical subgroups of children with JIA and wrist involvement, adding time-intensity-curve shape analysis to the outcome measures and performing movement registration to improve image quality. The hypothesized more aggressive enhancement patterns visualized by time-intensity-curve shapes were not significantly more present in children with clinically active disease. Beforehand, the time-intensity-curve shapes were thought to be discriminative, as previous studies in JIA and rheumatoid arthritis also showed aggressive enhancement patterns in active disease [23–25]. Time-intensity-curve shapes 3, 4 and 5, associated with a rapid enhancing curve in the early phase, were especially thought to be indicative of high vascularization and inflammation. The time-intensity-curve shape patterns showed, in general, a rather large overlap between clinically active and inactive disease, which makes them less likely to be good clinical discriminators. The lack of significant differences with respect to time-intensity-curve shapes is most likely to be attributed to a lack of power, caused by the small number of children with inactive disease requiring an MRI.

On the other hand, one conventional descriptive measures of dynamic contrast-enhanced MRI did prove to be indicative of clinically active disease, as there was a significant difference with the clinically inactive group. The added value of maximum enhancement for distinguishing clinically active versus clinically inactive disease in the JIA wrist is consistent with previous findings in the wrist affected by rheumatoid arthritis and the knee affected by JIA. However, these differences were significant on a group level; no value for an individual difference was shown in this study [8, 14, 23].

The validity of dynamic contrast-enhanced MRI and its potential relevance in JIA was further explored by the correlation with Juvenile Arthritis Disease Activity Score as a clinical measure for disease activity. The only measure reaching a moderate correlation was the enhancing volume, despite the lack of significant difference in enhancing volume between active and inactive disease. On the other hand, correlations between clinical measures and MRI are not expected to be very high, as both entities are considered more complementary than similar.

Feasibility of the chosen method for image acquisition with respect to the movement registration and positioning of the arm proved to be good. The movement registration algorithm visibly improved the image quality by correcting for possible movement occurring between scans. The risk of motion artifacts was also reduced by a relatively comfortable position for patients in our center undergoing MRI of the wrist in an open-bore 1.0-T magnet with the arm along the side of the body. Some other MRI systems might suffer from more motion artifacts, due to the required artifact-prone Superman-like position. In this case, the movement registration algorithms would be even more valuable. On the other hand, the arm along the side of the body also has its limitations, especially with respect to higher susceptibility to fold-over artifacts if the arm is not moved far away enough from the body.

This most important limitation of this study is the fact that the clinical subgroups were unequally divided. Very few children with clinically inactive disease and previous wrist involvement had a clinical indication for MRI in our patient clinics, which led to a low number of children with inactive disease in our study and, therefore, underpowered statistical analysis. This equal distribution of subgroups deserves attention in future studies. Furthermore, the field strength of 1.0 T, chosen because of the geometrical advantage of the open scanner, could have influenced image quality (the signal intensity and temporal resolution is, in general, lower than on equivalent protocols in higher-field cylindrical scanner) and the results. Future dynamic contrast-enhanced MRI studies are preferably performed on machines with a field strength of 1.5 T or higher.

## Conclusion

This study indicates that dynamic contrast-enhanced MRI is promising as a functional biomarker for children with JIA with wrist involvement and that descriptive parameters of dynamic contrast-enhanced MRI are better associated with clinical disease status of the wrist than time-intensity-curve shape analysis. Currently, the clinician treating children with JIA can use the maximum enhancement and enhancing volume as markers more suggestive of clinically active disease. Further implementation of dynamic contrast-enhanced MRI in clinical practice requires investigation of the possibilities of automated post-processing of the images, as well as the assessment of the added value for the individual child with JIA with respect to treatment and clinical course. This can be achieved by determining whether dynamic contrast-enhanced MRI is, for example, predictive of therapy response or long-term outcome.

## Electronic supplementary material

Below is the link to the electronic supplementary material.

ESM 1 (DOC 44 kb)

ESM 2 (DOC 74 kb)
