# Supplementary material for: Dynamic contrast-enhanced magnetic resonance imaging of the wrist in children with juvenile idiopathic arthritis
Source: Pediatr Radiol. 2016 Dec 12;47(2):205–13. doi: 10.1007/s00247-016-3736-2 (PMC5250661; doi:10.1007/s00247-016-3736-2)
Supplement: Supplementary file 1 — (DOC 44 kb) [file 247_2016_3736_MOESM1_ESM.doc]

**Table 1 –** Image acquisition characteristics of studies using DCE-MRI in the wrist

| **Author** | **Year** | **Population** | **n** | **MRI** | **Protocol**‡ | **Voxel** (mm) | **Time (rep.)** |
| --- | --- | --- | --- | --- | --- | --- | --- |
| Axelsen | 2014 | RA, healthy | 40 | 1.0T | Cor SE 40/12 105x140 | 0.5 x 0.5 x 3.0 | 10s (20) |
| Boesen | 2012 | RA | 54 | 0.2T | Cor GE 60/6 180x180 | 0.7 x 0.7 x 4.0 | 10s (30) |
| Boesen | 2011 | RA, PsA, healthy | 54 | 0.2T | Ax SE 100/16 150x150 | 0.9 x 1.2 x 5.0 | 18s (20) |
| Cimmino | 2003 | RA, healthy | 41 | 0.2T | Ax SE 100/16 150x150 | 0.9 x 1.2 x 5.0 | 18s (20) |
| Cimmino | 2005 | RA, PsA, healthy | 102 | 0.2T | Ax SE 100/16 150x150 | 0.9 x 1.2 x 5.0 | 18s (20) |
| Cimmino | 2012 | PsA | 7 | 0.2T | Ax SE 100/16 150x150 | 0.9 x 1.2 x 5.0 | 18s (20) |
| Hodgson | 2007 | RA, healthy | 11 | 3.0T | 3D SPGR 4.5/2 204x102 | 0.8 x 0.8 x 1.0 | 13s (24) |
| Huang | 2000 | RA | 42 | 1.5T | Cor SPGR 150/9.1 120x120 | 0.5 x 0.5 x 3.0 | 42s (9) |
| Kalden-Nemeth | 1997 | RA | 53 | 1.5T | Cor GE 0.27/10 200x200 | 0.9 x 0.8 x 3.0 | 6s (77) |
| Malattia | 2010 | JIA | 12 | 1.5T | Cor FFE 6/1.7 | - | 5s (40) |
| Meier | 2014 | RA | 28 | 3.0T | Cor GE 3.83/1.34 185 x 220 | 1.0 x 0.86 x 2.5 | 2.4s (139) |
| Navalho | 2012 | RA | 18 | 3.0T | 3D VIBE | 1.0 x 1.0 x 1.1 | 28s (8) |
| Ostergaard | 1996 | RA | 26 | 1.5T | Ax FLASH 40/12 120x150 | 0.5 x 0.6 x 5.0 | 10s (30) |
| Palosaari | 2004 | RA | 28 | 0.23T | Cor GE 30/10 160x160 | 1.3 x 6.3 x 2.0 | 69s (4) |
| Schwenzer | 2010 | RA, PsA | 45 | 3.0T | FLASH GE 3.91/1.45 | 0.8 x 0.8 x 0.8 | 10s (16) |
| Tam | 2007 | RA | 19 | 1.5T | --- | --- x --- x 4.0 | 10s (20) |
| Wojciechowski | 2013 | RA | 46 | 0.2T | Cor GE 60/6 180x180 | 0.7 x 0.7 x 5.0 | 10s (30) |

‡=direction, type of sequence, TR/TE and FOV; Ax=axial; Cor=coronal; EJR=European Journal of Radiology; FLASH=fast low angle shot; FFE=fast field echo; FOV=field of view (mm); GE=gradient-echo; PsA=psoriatic arthritis; RA=rheumatoid arthritis; Rheum=Rheumatology (journal); SE=spin-echo; SPGR=spoiled gradient echo; TE=echo time (ms); TR=repetition time (ms); VIBE=volume-interpolated breath hold examination
