# Supplementary material for: Dynamic contrast-enhanced magnetic resonance imaging of the wrist in children with juvenile idiopathic arthritis
Source: Pediatr Radiol. 2016 Dec 12;47(2):205–13. doi: 10.1007/s00247-016-3736-2 (PMC5250661; doi:10.1007/s00247-016-3736-2)
Supplement: Supplementary file 2 — (DOC 74 kb) [file 247_2016_3736_MOESM2_ESM.doc]

**Table 2** – Imaging analysis characteristics of studies using DCE-MRI in the arthritic wrist (i.e. both rheumatoid arthritis and juvenile idiopathic arthritis)

|  |  | **Movement**  **registration** | | **Drawing method ROI** | | **Shape of ROI** | | **No. slices with ROI** | | **Type of slice for ROI** | | |  | **Outcome measures** | |  |
| --- | --- | --- | --- | --- | --- | --- | --- | --- | --- | --- | --- | --- | --- | --- | --- | --- |
| Author | Year | Yes | No | Automatic | Manual | Anatomy | Standard | 1, 2 or 3 | All | Gd | ME | IRE | MIP/SBT | Classic | Curve type | Ph.kinetic |
| Axelsen | 2014 |  |  |  |  |  |  |  |  |  |  |  |  |  |  |  |
| Boesen | 2012 |  |  |  |  |  |  |  |  |  |  |  |  |  |  |  |
| Boesen | 2011 |  |  |  |  |  |  |  |  |  |  |  |  |  |  |  |
| Cimmino | 2003 |  |  |  |  |  |  |  |  |  |  |  |  |  |  |  |
| Cimmino | 2005 |  |  |  |  |  |  |  |  |  |  |  |  |  |  |  |
| Cimmino | 2012 |  |  |  |  |  |  |  |  |  |  |  |  |  |  |  |
| Hodgson | 2007 |  |  |  |  |  |  |  |  |  |  |  |  |  |  |  |
| Huang | 2000 |  |  |  |  |  |  |  |  |  |  |  |  |  |  |  |
| Kalden-Nemeth | 1997 |  |  |  |  |  |  |  |  |  |  |  |  |  |  |  |
| Malattia | 2010 |  |  |  |  |  |  |  |  |  |  |  |  |  |  |  |
| Meier |  |  |  |  |  |  |  |  |  |  |  |  |  |  |  |  |
| Navalho | 2012 |  |  |  |  |  |  |  |  |  |  |  |  |  |  |  |
| Ostergaard | 1996 |  |  |  |  |  |  |  |  |  |  |  |  |  |  |  |
| Palosaari | 2004 |  |  |  |  |  |  |  |  |  |  |  |  |  |  |  |
| Schwenzer | 2010 |  |  |  |  |  |  |  |  |  |  |  |  |  |  |  |
| Tam | 2007 |  |  |  |  |  |  |  |  |  |  |  |  |  |  |  |
| Wojciechowski | 2013 |  |  |  |  |  |  |  |  |  |  |  |  |  |  |  |

ROI=region of interest; Gd=pre- and post-gadolinium images; ME=maximum enhancement; IRE=initial rate of enhancement; MIP=maximum intensity projection; SBT=subtraction images; Ph.kinetic=pharmacokinetic
